# Multiplexed Autoantibody Signature for Serological Detection of Canine Mammary Tumours

**DOI:** 10.1038/s41598-018-34097-0

**Published:** 2018-10-25

**Authors:** Shahid Hussain, Sonal Saxena, Sameer Shrivastava, Richa Arora, Rajkumar James Singh, Subas Chandra Jena, Naveen Kumar, Anil Kumar Sharma, Monalisa Sahoo, Ashok Kumar Tiwari, Bishnu Prasad Mishra, Raj Kumar Singh

**Affiliations:** 1 Division of Veterinary Biotechnology, ICAR-Indian Veterinary Research Institute [Deemed University] Izatnagar, Bareilly, UP India; 2 Division of Veterinary Surgery, ICAR-Indian Veterinary Research Institute [Deemed University], Izatnagar, Bareilly, UP India; 3 Division of Veterinary Pathology, ICAR-Indian Veterinary Research Institute [Deemed University], Izatnagar, Bareilly, UP India

## Abstract

Spontaneously occurring canine mammary tumours (CMTs) are the most common neoplasms of female unspayed dogs and are of potential importance as models for human breast cancer as well. Mortality rates are thrice higher in dogs as compared to humans with breast cancer, which can partly be attributed to lack of diagnostic techniques for their early detection. Human breast cancer studies reveal role of autoantibodies in early cancer diagnosis and also the usefulness of autoantibody panels in increasing the sensitivity, as well as, specificity of diagnostic assays. Therefore, in this study, we took advantage of high-throughput Luminex technique for developing a multiplex assay to detect autoantibody signatures against 5 canine mammary tumour-associated autoantigens (TAAs). These TAAs were expressed separately as fusion proteins with halo tag at the N-terminus, which allows easy and specific covalent coupling with magnetic microspheres. The multiplex assay, comprising a panel of candidate autoantigens (TPI, PGAM1, MNSOD, CMYC & MUC1) was used for screening circulating autoantibodies in 125 dog sera samples, including 75 mammary tumour sera and 50 healthy dog sera. The area under curve (AUC) of the combined panel of biomarkers is 0.931 (p < 0.0001), which validates the discriminative potential of the panel in differentiating tumour patients from healthy controls. The assay could be conducted in 3hrs using only 1ul of serum sample and could detect clinical cases of canine mammary tumour with sensitivity and specificity of 78.6% and 90%, respectively. In this study, we report for the first time a multiplexed assay for detection of autoantibodies in canine tumours, utilizing luminex technology and halo-tag coupling strategy. Further to the best of our knowledge, autoantibodies to CMYC and MUC1 have been reported for the first time in canines in this study.

## Introduction

The immune system responds to low, usually undetectable levels of tumour-associated autoantigens (TAAs) by mounting a very specific antibody response, providing opportunities for early cancer detection^1–4^. Autoantibodies against TAAs have previously been identified in cancers of the colon^5^, breast^6^, lung^7^, ovary^8^, prostate^3^, head & neck^9^, oesophagus^10^ and pancreas^11^. Interest in the use of anti-TAA antibodies as serological biomarkers for cancer diagnosis and prognosis derives from the recognition that these autoantibodies are either absent or present in very low titres in healthy individuals. Their measurement in serum is minimally invasive and cost effective using established technologies. Their persistence and stability in the serum of cancer patients is an advantage over other potential markers, including the TAAs themselves, which are released by tumours, but are rapidly degraded or cleared after circulating in the blood for a limited time^12^. Thus, cancer-associated autoantibodies might be regarded as reporters, identifying aberrant *de novo* or dysregulated cellular mechanisms in tumorigenesis^2,3^ and holds great potential as an ideal cancer screening and diagnostic tool^13,14^. However, most autoantibodies discovered so far have limited diagnostic value alone as frequencies of autoantibodies specific for a particular TAA in a cancer population are often low, ranging from 10–30%^14–16^. Further, a single antibody may not discriminate between cancer types and may arise as a consequence of molecular events associated with cancer or other diseases. Therefore, multiplexing of autoantibody biomarkers is required to increase the sensitivity and specificity of the diagnostic assays. Microsphere-based suspension array technology based upon the Luminex® xMAP™ system, offers a new multiplexing platform for high-throughput analyte detection^17–19^. Some benefits of suspension array technology over conventional immunoassays include rapid data acquisition (few hours), excellent sensitivity and specificity, multiplexed analysis capability and low sample requirement. Canine mammary tumour (CMT) is the most common malignancy of unspayed female dogs leading to at least three times higher mortality rates than human breast cancer^20^. Most of the techniques currently used for CMT diagnosis are invasive and provide diagnosis when the tumour burden has crossed a threshold level. In humans, a number of autoantibody biomarkers have been identified and panel assays against breast cancer^21–23^, lung cancer^24^, ovarian cancer^25^ etc., have been developed, demonstrating increased sensitivity and specificity for cancer detection. However, in dogs very few autoantibody biomarkers associated with cancer have been identified and no studies have been conducted so far to evaluate diagnostic utility of autoantibody panels. Considering the significance of autoantibodies as markers for early cancer detection, and multiplexing as a strategy to increase sensitivity, the present study was designed to develop a multiplex autoantibody based panel assay for diagnosis of CMT in dogs. The assay was developed using commercially available magnetic micropsheres (MagPlex® microspheres) which have advantages of maximal recovery during handling and facilitates assay automation. The candidate autoantigens chosen for developing the five-plex assay were triose phosphate isomerase (TPI), manganese-superoxide dismutase (MNSOD), phosphoglycerate mutase1 (PGAM1), avian myelocytomatosis viral oncogene homolog (CMYC) and mucin1 glycoprotein (MUC1). TPI and PGAM1 are glycolytic enzymes involved in the glycolysis metabolism, which plays a critical role in supply of ATP to the neoplastic cells^26^. TPI & PGAM1 coordinates glycolysis and carbohydrate biosynthesis to promote cancer growth and metastasis. Parallel to glycolytic metabolism, high level of MNSOD is expressed by the cancer cells to cope up higher oxidative stress and MNSOD is now considered as a potential marker for tumour progression & metastasis^27–29^. MUC1 is a transmembrane mucin, which is aberrantly overexpressed in over 90% of breast tumours and is well studied as a diagnostic marker for metastatic progression^30–32^. CMYC (MYC) is a transcription factor regulating more than 15% of human genes, involved in cell proliferation, differentiation, adhesion, apoptosis, and migration^33^. Deregulated expression of CMYC has been found in multiple cancer types, including human breast cancer^34^ and canine mammary cancer^35^. Thus in recent years, MNSOD^36^, TPI^37^, PGAM1^38^, MUC1^32^ and CMYC^34^ have emerged as new targets for cancer diagnosis and therapy. TPI, PGAM1, MNSOD were selected for this study based upon the studies of Zamani-Ahmadmahmudi *et al*.^39^, who by serological proteome analysis (SERPA) identified these as potential autoantigens with significantly higher immunoreactivity in canine mammary cancer sera samples. Also in our lab, we have demonstrated diagnostic potential of TPI, MNSOD, PGAM1, MUC1 & CMYC autoantibodies in canine mammary tumours by ELISA (Supplementary Table S8). The autoantibodies to these five antigens were found to be present in higher frequency in canine mammary cancer sera, as compared to healthy dog sera. Further autoantibodies to these five antigens have been identified by various researchers as potential biomarkers in human cancers also. Yang and colleagues identified TPI and MNSOD panel to have potential for diagnosis of early-stage cancers with 47% sensitivity and 90% specificity^40^. Using immunoproteomic techniques, TPI autoantibodies were demonstrated in high frequency of human breast cancers^41,42^. Tamesa *et al*.^41^ detected autoantibodies against TPI in 90% of the human breast cancer sera samples. High-titre circulating anti-Mucin1 (MUC1) antibodies have been reported in human breast tumor patients^43^. In other study, autoantibodies against MNSOD have been demonstrated in 37.5% of human breast cancer sera samples^44^. Similarly, researchers have also reported presence of anti-myc antibodies as diagnostic marker in early stage human cancer patients^45^. Thus autoantibodies against CMYC^45^, MUC1^43,46^, MNSOD^40^, TPI^40^ have been implicated in human cancers to have relevance for early cancer diagnosis, however the diagnostic suitability of these biomarkers as a panel has not been attempted previously. Therefore, in this study microsphere based multiplex assay was developed for detection of TPI, MNSOD, PGAM1, MUC1 & CMYC autoantibody signatures in dogs suffering from CMT. A total of 125 dog sera samples, including 75 from clinical cases of canine mammary tumour (CMT) and 50 healthy dogs, were analysed to assess the diagnostic potential of the panel assay.

## Materials and Methods

### Instruments and reagents for multiplex assay

Carboxy-functionalized MagPlex microspheres (#MC10028, MC10035, MC10045, MC10054, MC10064) were purchased from Bio-Rad laboratories India Pvt. Ltd, Gurgaon. The multiplexed assay was analysed on Bio-Plex 200 system, which is a suspension array system with two lasers: the classification laser (635 nm excitation), for identifying the bead signatures, and the reporter laser (532 nm excitation) for detecting the target. 1-Ethyl-3-(3-dimethylaminopropyl)-carbodiimide hydrochloride (EDC, *#*E6383), N-hydroxysulfosuccinimide (sulfo-NHS, #56485), anti-dog IgG-biotin (#SAB3700111), streptavidin-conjugated phycoerythrin (SA-PE, #42250), phosphate buffered saline (PBS, #P4417), bovine serum albumin (BSA, #A2153) and tween-20 (#P1379) were purchased from Sigma-Aldrich (St. Louis, USA). Rabbit polyclonals against PGAM1 (#SC-292579), TPI (#SC-30145), MNSOD (#SC-30080), CMYC (#SC-788) were purchased from SantaCruz (SantaCruz, USA). Rabbit polyclonal against MUC1 (#AV41445) was purchased from Sigma-Aldrich (St. Louis, USA). Anti-halo tag antibody, halo tag Amine (O4) ligand (#P-6741) and pH6HTN His_6_HaloTag® T7 vector (#G7971) were purchased from Promega (Promega, USA).

### Sera and tumour tissue samples

Sera and tissue samples were collected from dogs that were presented at the “Referral Veterinary Polyclinic”, ICAR-Indian Veterinary Research Institute, Izatnagar, U.P. India. Total 125 dog sera samples were collected including 75 from cases of canine mammary tumour (CMT) and 50 healthy female dogs. Healthy dogs were examined for the absence of any disease by clinico-physical & hematological parameters such as CBC, LFT, KFT and urinalysis. Tumour tissues obtained from tumour biopsy/surgical resections were fixed in neutral buffered formalin for histopathological analysis and immunohistochemistry. Histopathological classification and grading of the paraffin embedded tissue sections was done as per Goldschimdt *et al*.^47^. Based on histopathological analysis of hematoxylin and eosin stained tumour tissue sections, tissues were classified as malignant and benign. Serum samples were collected within 1 week of the first biopsy-proven mammary tumour diagnosis, and prior to removal of the tumour by a surgical procedure or start of chemotherapy regime. [Details for histopathological classification & grading of tumour tissues are provided in Supplementary Table S1. Representative photographs for histopathological analysis of tumour tissues are also shown in Supplementary Fig. S1].

### Immunohistochemistry

Tissue samples were fixed, paraffin-embedded and then cut into 5 µm sections. Sections were mounted on 3-aminopropyl-triethoxy-silane (APTES) coated slides, and air-dried overnight at 37 °C. Prepared slides were deparaffinized in three washes of xylene (10 min each), and rehydrated in graded concentrations of ethanol. The slides were then washed with PBS and blocked with 5% horse serum for 1 hour and further steps were carried as per SuperPicture™ polymer detection Kit (Thermofischer Scientific, USA) with slight modifications. Briefly, 100 μl of quenching solution was added to completely cover the tissue sections for 5 minutes, followed by three rinses in PBS. The sections were then incubated overnight with 1:50 dilution of protein specific antibody. [PGAM1 (#SC-292579, Santa Cruz USA), TPI (#SC-30145, Santa Cruz USA), MNSOD (#SC-137254, Santa Cruz USA), CMYC (#SC-788, Santa Cruz USA), MUC1 (#AV41445, Sigma Aldrich USA)]. Post incubation, slides were washed with PBST (PBS with 0.05% Tween-20) and HRP polymer conjugate was added to completely cover the sections for 30 minutes. DAB chromogen was then added and incubated for 5 minutes followed by washing in distilled water. The sections were counter-stained using Mayer’s hematoxylin for 10–15 seconds. The control used for IHC was process control/isotype control wherein IHC was performed using isotype-specific immunoglobulins at the same concentration as of primary antibody, with rest of the procedure similar to test. To determine overexpression of the candidate biomarkers, IHC staining was compared between CMT and healthy mammary tissues.

### Real-time PCR

The primers were designed for qRT-PCR analysis using the Integrated DNA technologies- Primer Quest Tool. The details of primers sequences used for the study are mentioned in Table 1. Total RNA was extracted from tumour tissues preserved in RNAlater (Ambion, Life Technologies) as described previously^48^. The cDNA was synthesized using Revert Aid First Strand cDNA synthesis kit (Thermofischer Scientific, USA) according to the manufacturer’s instructions and qRT-PCR was performed using Applied Biosystems 7500 Fast system as described previously^48^. Gene expression in each sample was normalized against the expression of housekeeping gene (β-actin). The relative expression of each sample was calculated using the 2^−ΔΔCT^ method with healthy mammary tissue as calibrator and log_2_ fold change was plotted.

**Table 1:** Primer sequences for qRT-PCR analysis of target genes.

Gene	Primer	Sequence
PGAM1	Forward	GACCATCCCTTCTACAGCAAC
	Reverse	GGCAATGGTATCCTTCAGACTC
MNSOD	Forward	GGACAAACCTGAGCCCTAAG
	Reverse	AAGCCAACCCCAACCTG
TPI	Forward	TCAGAGCACCCGTATCATTTAC
	Reverse	GGGCTTATTGTTTGGCGTTG
CMYC	Forward	ACTCTCTGCTCTCCTCGG
	Reverse	TTCTTCGTCCTCTTGTTCTTCC
MUC1	Forward	GACAAGACCTCCTATCGCTG
	Reverse	AGAATACACCAAGACGCCC

### Generation of recombinant expression vectors and expression of halo-tagged fusion proteins

One microgram of the total RNA sample was used to synthesize cDNA using Revertaid cDNA synthesis kit (ThermoScientific, USA) according to the manufacturer’s protocol. Primers were designed for amplification of immunodominant regions from the target genes using Primer Express Software v 3.0.1(Life Technologies, USA). (Primer details are provided in Supplementary Table S2). The PCR products were cloned individually in pH6HTN His_6_HaloTag® T7 prokaryotic expression vector (Promega, USA). The recombinant clones were confirmed by PCR, restriction endonuclease analysis and plasmid DNA sequencing. Recombinant plasmids were transformed individually in *E*.*coli* KRX cells (Promega, USA). The transformed colonies were inoculated into LB medium containing 100 ug/ml ampicillin, and induced using 0.1% rhamnose and 1 mM isopropy-β-D-thiogalactopyranoside (IPTG) and purified by affinity chromatography using AKTA pure 25 M Fast Performance Liquid Chromatography (FPLC) (GE healthcare, Sweden) as described earlier^49^. The purified proteins were characterized by SDS-PAGE and western blotting.

### Immobilization of recombinant proteins on MagPlex microspheres

**Figure 1:**
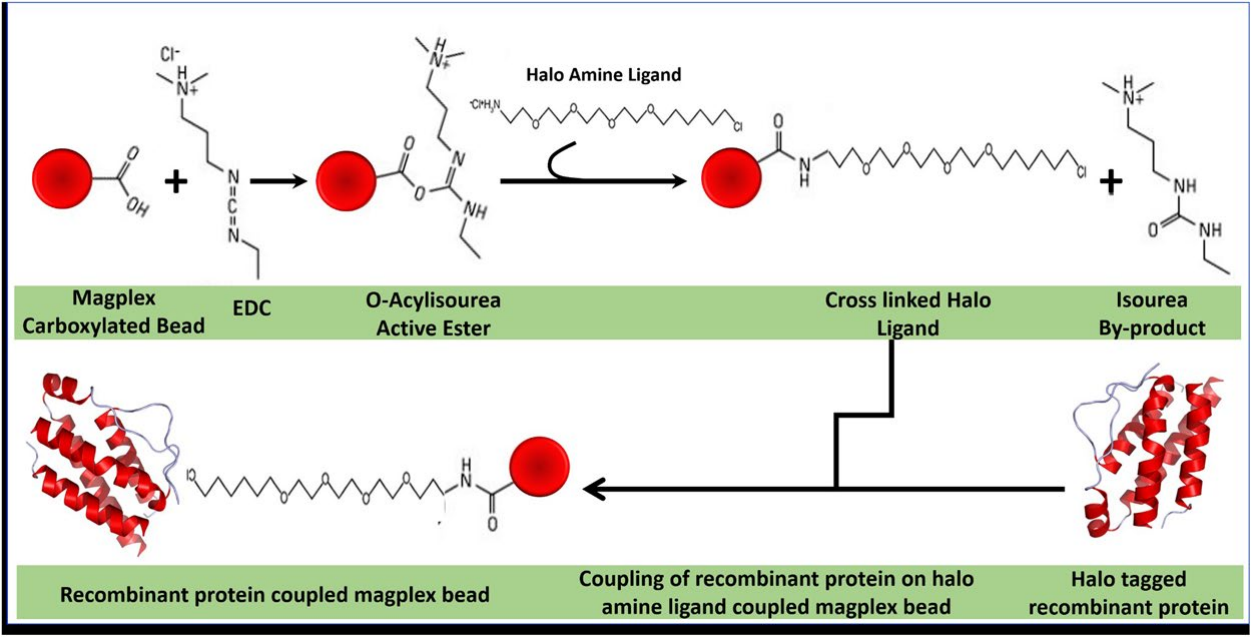
Strategy for coupling of halotagged recombinant TAAs on MagPlex microspheres. Halotagged recombinant proteins (HTP) were immobilized on MagPlex microspheres (BioRad, USA) by first conjugating HaloTag Amine (O4) ligand to the microspheres, using amine coupling strategy. The halo ligand immobilized magnetic micropsheres were then coupled to HTP. The coupling between HTP and HaloTag® Amine (O4) ligand was based upon the nucleophilic attack by the chloroalkane to Asp 106 in the HTP, resulting in the formation of an ester bond between the HaloTag ligand and the recombinant protein. HTP contains a critical mutation in the catalytic triad (His 272 to Phe) so that the ester bond formed between HTP and HaloTag ligand cannot be further hydrolysed.

Covalent immobilization of the expressed recombinant proteins on MagPlex microspheres was accomplished using halotag coupling strategy. For coupling, the recombinant TAAs were expressed as a fusion protein with an engineered haloalkane dehalogenase (Halo protein), employing pH6HTN His_6_HaloTag® T7 prokaryotic expression vector (Promega, USA), as described earlier. The strategy for immobilization of halo-tagged recombinant TAAs on MagPlex microspheres is depicted in Fig. 1. Halo-tagged recombinant proteins were immobilized on MagPlex microspheres (BioRad, USA) by first conjugating HaloTag amine (O4) ligand to the beads, using amine coupling strategy. A total of 1.25 × 10^6^ beads were conjugated with 0.2 mg of halo amine (O4) ligand as per the protocol of Jia *et al*.^50^. The microspheres were suspended in 100 mmol/L solution of 2-(*N*-morpholino)ethanesulfonic acid (MES) [pH 6.0], containing 5 mg/mL 1-ethyl-3-(3-dimethylaminopropyl) carbodiimide and 0.2 mg halo amine ligand. After incubation for 2 hrs, the microspheres were washed and re-suspended in 100 mmol/L MES (pH 4.5), and stored at 4 °C. Further the halo protein tagged recombinant TAAs were immobilized on the halo amine ligand coupled microspheres. Different MagPlex bead regions were coupled overnight on rotator at 4 °C with 5 µg of different HaloTag recombinant proteins. Post incubation, beads were washed in bead wash buffer (BioRad, USA) and re-suspended in 150 µl of storage buffer (BioRad, USA). The coupling between halo-tagged protein (HTP) and HaloTag® amine (O4) ligand is based on the nucleophilic attack by the chloroalkane to Asp 106 in the HTP resulting in the formation of an ester bond between the HaloTag ligand and the HTP. HTP contains a critical mutation in the catalytic triad (His 272 to Phe) so that the ester bond formed between HTP and HaloTag ligand cannot be further hydrolysed. Therefore, the bonding is highly specific and essentially irreversible, resulting in formation of a complex on microspheres that is highly stable even under stringent conditions.

### Validation and standardization of coupling on MagPlex microspheres

Considering that coupled microspheres could be cleared during the washing steps, concentration of coupled microspheres was determined by performing a total bead count for each region using Neubauer counting chamber. For confirmation of coupling of recombinant proteins on the microspheres, rabbit polyclonal antibodies against different TAAs were used. The protein coupled microspheres were resuspended by brief vortexing for approximately 20 seconds and a working microsphere mixture was prepared by diluting the coupled microsphere stocks to a final concentration of 1000 microspheres per well in PBS buffer. Tenfold serial dilutions of each antibody were incubated with different protein conjugated microspheres in a 96-well plate for 1 hour. After washing with wash buffer [PBS with 1% BSA & 0.02% Tween-20], the microspheres were incubated with biotinylated secondary antibody (4 µg/ml) for 1 hr. The resulting complex was washed again and incubated with Streptavidin-Phycoerythrin (S-PE) for 10 minutes. Finally, the micropsheres were washed and resuspended in assay buffer [PBS with 0.1% BSA and 0.02% Tween] and read on Bio-plex 200 system (Biorad, USA). Background corrected mean florescence intensity (MFI) was recorded, which indicated the binding signal. To ensure that individual protein coupled beads reacts with corresponding antibodies only, the specificity of the multiplex assay was tested by equally mixing different protein coupled microspheres (PGAM1, TPI, MNSOD, MUC1 and CMYC) and distributing into a 96 well plate and reacting with individual protein specific rabbit polyclonal antibodies on the multiplex array. To compare uniplex and multiplex system, 12 randomly selected sera samples were diluted 1:200 in PBS/1% BSA. The serum samples were added in duplicates to wells containing single protein coupled microspheres, as well as, mixture of different protein coupled microspheres. MFI values for the randomly selected sera samples corresponding to the given biomarkers were then compared between the uniplex and multiplex systems. For determining the assay parallelism, known quantities of polyclonal antibodies against TAAs were spiked in healthy dog sera. Slopes obtained from spike concentration-response curve in healthy dog serum were compared with that of the standard polyclonal antibody concentration response curve using four-parameter logistic (4-PL) curve fitting. To assess, the assay performance, the reproducibility of the assay was examined by determining the inter-assay, as well as, intra-assay coefficients of variation (%CV). Intra-assay %CV values were calculated from the MFIs of all the three replicates in a single plate at each standard dilution point from representative serum samples. For calculation of inter-assay %CV, the antibody concentrations corresponding to the representative sera samples from three independent plate measurements were taken into consideration.

### Detection of autoantibody biomarkers in dog sera

**Figure 2:**
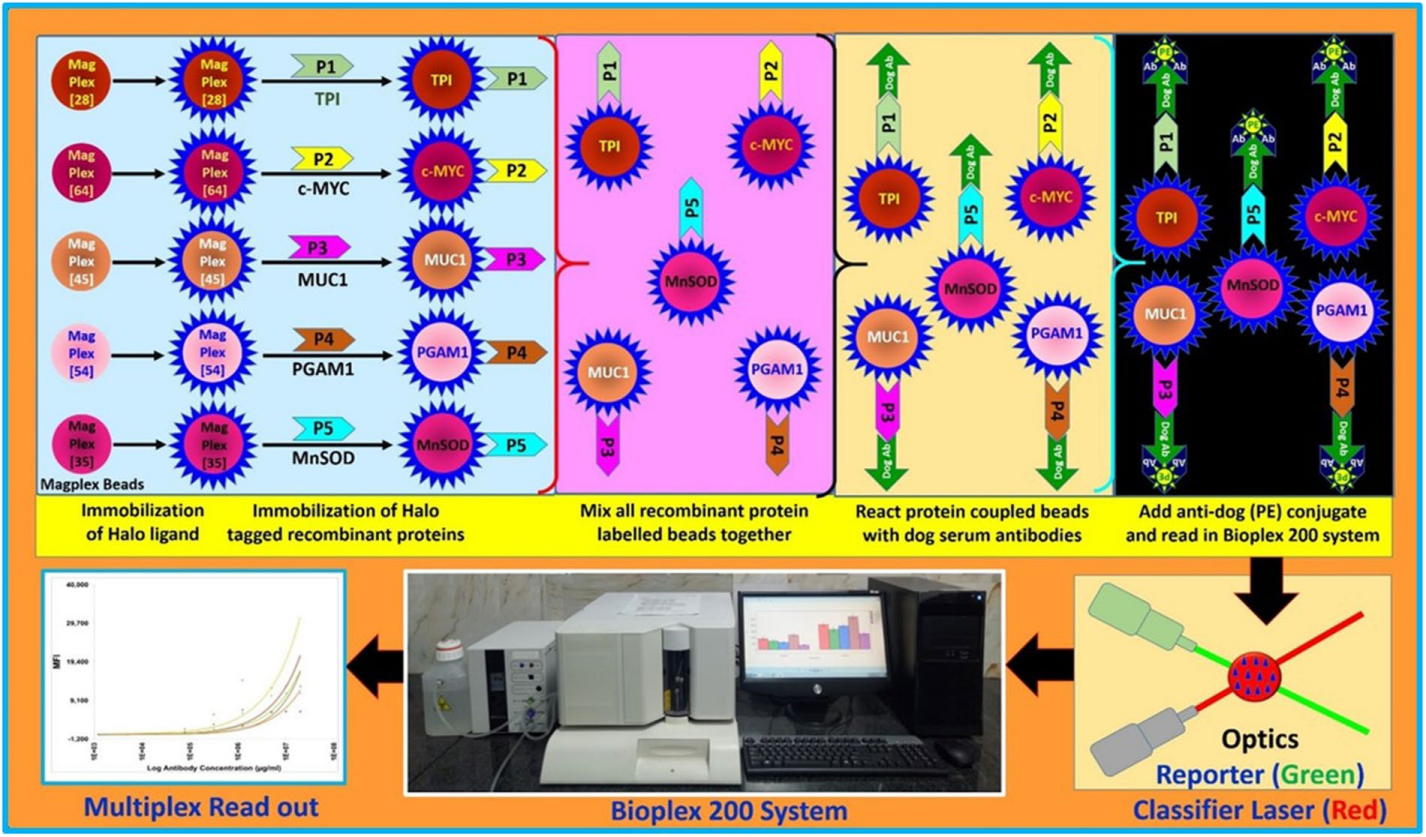
Schematic representation of strategy for developing multiplex immunoassay for detecting autoantibody biomarkers in dog sera samples.

Different microspheres conjugated with recombinant proteins were mixed together and distributed into a 96-well plate (1000 microspheres/well) to detect corresponding autoantibodies in dog serum. The serum samples were diluted 1:200 in PBS with 1% BSA and incubated with the microspheres for 1 hour with shaking. Post incubation, microspheres were washed using wash buffer. Further, biotinylated anti-dog antibody [4 µg/ml] was added to the microsphere complex and incubated for 1 hour followed by washing with wash buffer. Then streptavidin-PE was added to the microspheres for 10 minutes. Finally, microspheres were washed, resuspended in 100 µl of the assay buffer and fed through the Bio-Plex™ 200 instrument following the manufacturer’s instructions. The instrument was programmed to read MagPlex microspheres in regions 028, 035, 045, 054 and 064 for TPI, MNSOD, MUC1, PGAM1 and CMYC coupled microspheres, respectively. A minimum of 100 events per microsphere were read and the median value obtained for each reaction event per microsphere set was recorded. Unless otherwise stated, all samples were analysed in duplicate and average readings were taken into consideration. MFI values for all the samples were corrected for background levels. Schematic representation of strategy for developing multiplex immunoassay for detecting autoantibody biomarkers in dog sera samples is displayed in Fig. 2.

### Statistical analysis

As background fluorescence intensity between microsphere sets can vary, the background values for the limit-of-detection were measured specifically for the capture microsphere sets used for the assay and all MFI values were subjected to background correction. The correlation coefficients between uniplex and multiplex systems, as well as, between different autoantibody biomarkers were evaluated using Pearson’s correlation coefficient (r). The data sets were tested for normal distribution and found not to be normally distributed. Therefore, statistical significance was tested using the Mann-Whitney-Test. Significance was defined at p < 0.05. Data analysis was done using Bio-Plex Data Pro software^TM^, GraphPad Prism (version 7.0) and MedCalc (version 14.8.1) softwares. Receiver Operator Characteristic (ROC) curve analysis^51^ was performed using MedCalc software (version 14.8). Herein, the sensitivity of a test is its ability to determine the tumour cases correctly and is defined as the probability that a test result will be positive when the disease is present (true positive rate). To calculate sensitivity, number of cases in the canine mammary tumour group that test positive [True positive (TP)] and negative [False negative (FN)] were first calculated, and then sensitivity was calculated as: TP/(TP + FN). The specificity of a test is its ability to determine the healthy cases correctly and is defined as the probability that a test result will be negative when the disease is not present (true negative rate). To calculate specificity, the number of cases in the non-diseased (Healthy) group that test positive [False positive (FP)] and negative [True negative (TN)] were determined, and then specificity was calculated as: TN/(TN + FP). The cut-off value designating positive reactions was chosen as the mean MFI of the healthy sera + 2 SD.

### Ethical Approval

All experimental procedures involving animals were in accordance with Breeding of and Experiments on Animals (Control and Supervision) Amendment Rules, Government of India, 2005. The experiments were conducted with due permission from the Institutional Animal Ethics Committee, Indian Veterinary Research Institute, Izatnagar and the Committee for the Purpose of Control and Supervision of Experiments on Animals (CPCSEA), Ministry of environment, forests and climate change, Govt of India.

## Results

### Overexpression of TPI, PGAM1, MUC1, MNSOD and CMYC in CMT tissues

**Figure 3:**
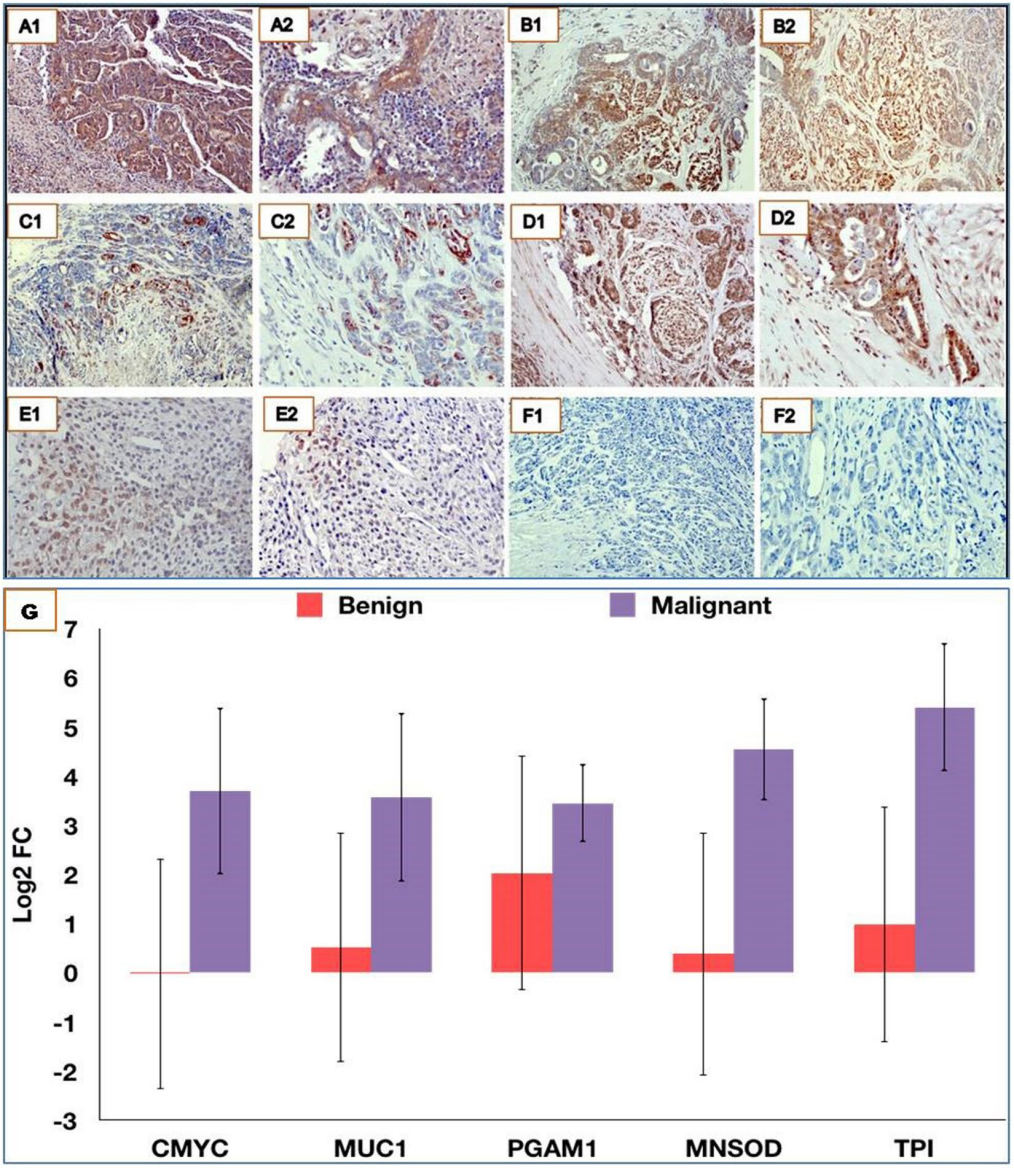
Overexpression of TPI, MNSOD, PGAM1, MUC1 & CMYC in canine mammary tumours. Immunohistochemical analysis at 10X (**A1**–**E1**) and 20X (**A2**–**E2**) revealed over expression of TPI (**A1**,**A2**), MNSOD (**B1**,**B2**), PGAM1 (**C1**,**C2**), MUC1 (**D1**,**D2**) and CMYC (**E1**,**E2**) in CMTs. F1(10X) & F2(20X) represent isotype controls. (**G**) qRT-PCR analysis also indicated overexpression of these biomarkers in CMT tissues.

Immunohistochemical analysis indicated the overexpression of TPI, MNSOD, PGAM1, MUC1 and CMYC in the mammary gland carcinoma (Fig. 3A–F). Upon close examination, it was observed that PGAM1 showed moderate immunostaining in the tubular epithelia, while a strong expression of PGAM1 was observed in the myoepithelia. TPI presented strong cytoplasmic and nuclear immunostaining in both tubular epithelium and myoepithelium. Expression of MUC1 was strong in cytoplasm of tubular epithelium and inflammatory cells. MNSOD showed strong membrane immunostaining of the tubular epithelium while CMYC showed moderate nuclear reactivity in the myoepithelial components.

qRT-PCR studies also revealed over-expression of PGAM1, MNSOD, TPI, MUC1 and CMYC in CMT tissues. A high level of concordance was observed between qRT-PCR and IHC for expression of MNSOD, TPI, MUC1 and CMYC in CMT tissues with correlation coefficient(r) values ranging between 0.79–0.86 for all the biomarkers. The relative gene expression levels for these five genes as calculated by 2^−ΔΔCT^ method in malignant (n = 10) and benign CMT tissues (n = 10), as compared to healthy mammary gland tissues (n = 2) are depicted in Fig. 3G. The mean expression levels of these genes (except PGAM1) were significantly (p < 0.05) higher in malignant CMT tissues in comparison to benign CMT tissues.

### Characterization of halo-tagged fusion proteins and confirmation of their immobilization on magnetic beads

**Figure 4:**
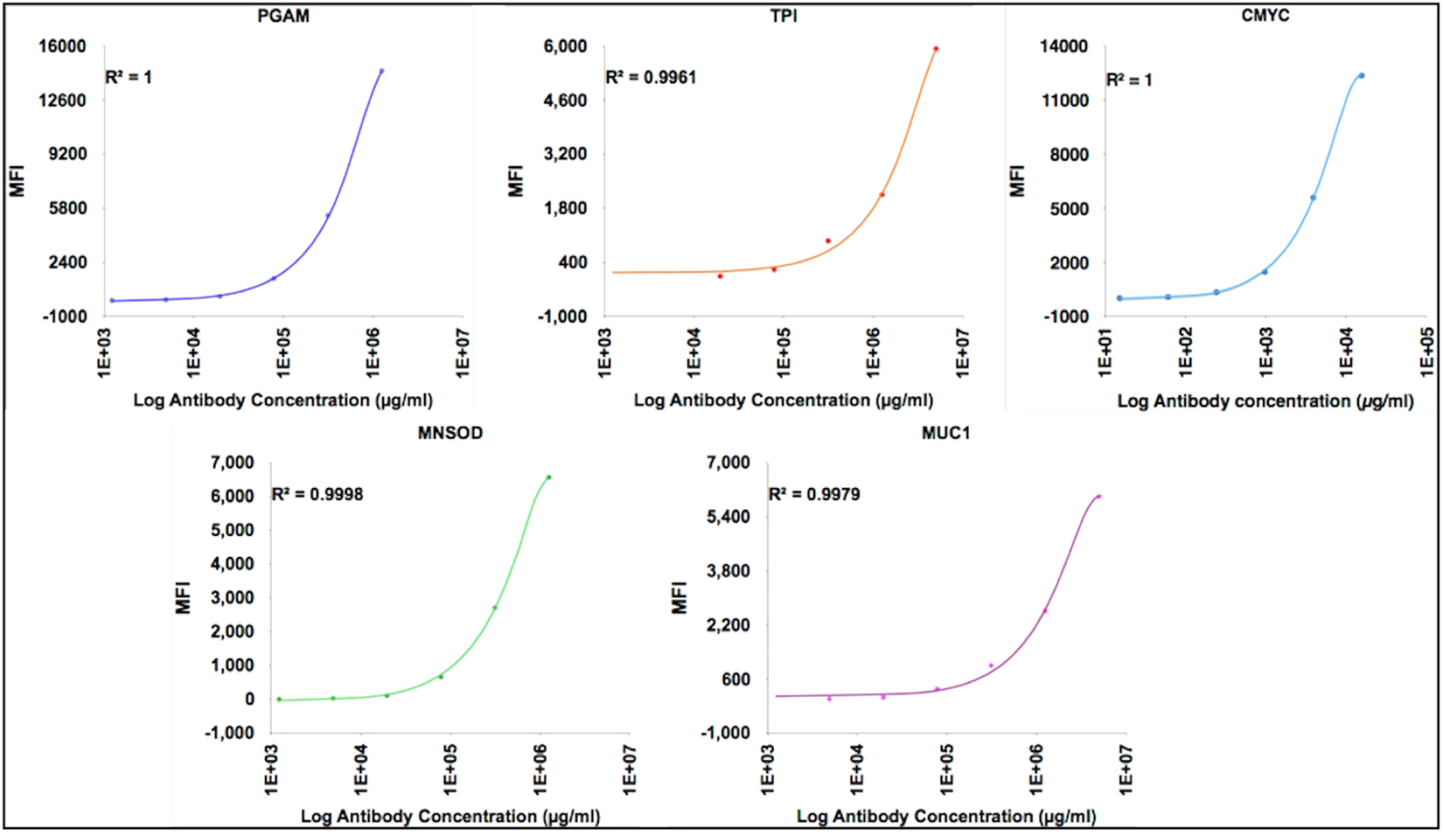
Validation of the coupling of MagPlex microspheres with the recombinant halotag fused TAAs. The protein coupled microspheres were reacted individually with increasing dilution of corresponding protein specific antibodies. The MFI signals generated by individual protein coupled microspheres increased with increasing concentration of antibodies in concentration dependent fashion.

The halo tagged recombinant TAAs were produced as described earlier. Immunoblot analysis of the recombinant proteins showed that the proteins reacted specifically with protein specific antibodies, as well as, with anti-halo antibodies confirming the presence of halo tag [SDS-PAGE and immunoblot analysis of recombinant TAAs are presented in Supplementary Fig. S2]. The immobilization of recombinant proteins on individual MagPlex microspheres was tested using protein specific antibodies. MFI signals corresponding to different concentration of antibodies (10-fold serial dilutions) were recorded. As depicted in Fig. 4, the MFI signals increased with the increasing concentration of antibodies. The regression coefficient (R^2^) values were calculated as 1, 0.996, 0.999, 0.997 and 1 for PGAM1, TPI, MNSOD, MUC1 and CMYC, respectively.

### Analysis of assay performance characteristics

**Figure 5:**
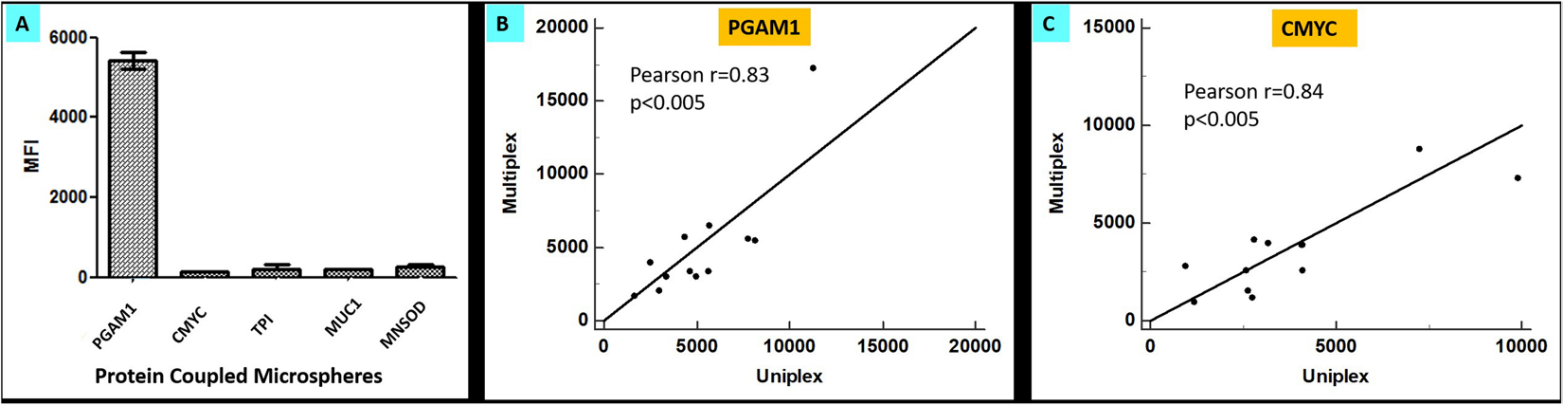
No cross-reactivity between individual protein-coupled microspheres in the multiplex system. (**A**) Multiplex system containing five microspheres coupled with TPI, MNSOD, PGAM1, MUC1 and CMYC was incubated with PGAM1 antibody, and only the PGAM1 microspheres reacted with anti-PGAM1 antibody, while other microspheres detected the background MFI values. (**B**) The signal correlation of the PGAM1 coupled microspheres in the singleplex and multiplex system. (**C**) Same as (**B**) but using CMYC coupled microspheres. The correlation between uniplex and multiplex systems was calculated by comparing the autoantibody signals generated from randomly selected serum samples (n = 12) by both the systems. The correlation coefficient (Pearson, r) was 0.83 for PGAM1 and 0.84 for CMYC (p < 0.005), indicating a significant correlation between the uniplex and multiplex systems.

The specificity of the multiplex assay was tested by equally mixing the individually protein coupled microspheres (PGAM1, TPI, MNSOD, MUC1 and CMYC) and distributing into a 96 well plate. Upon reacting with corresponding protein-specific antibodies on the multiplex array, the antibodies were only detected by their corresponding proteins bound on different bead regions and all other microspheres produced the background MFI signals, indicating no cross-reactivity between the individual protein-coupled microspheres. Figure 5A shows that only the PGAM1 microspheres reacted with the PGAM1 antibodies, resulting in significantly higher MFI values (p < 0.0001), than the background signals observed with the remaining four bead regions. Similar results were observed with other bead regions as well. Further, the correlation between uniplex and multiplex systems was calculated by comparing the autoantibody signals generated from randomly selected serum samples (n = 12) by both the systems. As shown in Fig. 5B,C, correlation coefficient (Pearson, r) was 0.83 for PGAM1 and 0.84 for CMYC (p < 0.005), indicating a significant correlation between the uniplex and multiplex systems.

Next, the assay parallelism was determined by spiking known quantities of polyclonal antibodies in healthy dog sera and then comparing slopes obtained from spike concentration-response curve in healthy dog serum with that of the standard polyclonal antibody concentration response curve, using four-parameter logistic (4-PL) curve fitting. The percentage differences in slope value with respect to standard curve were less than 18% for all the analytes in the panel.

To further assess, the assay performance, the reproducibility of the assay was examined by determining the inter-assay, as well as, intra-assay coefficients of variation (%CV). Intra-assay %CV values were calculated from the MFIs of replicates in a single plate at each standard dilution point from representative serum samples. All the analytes exhibited <7.4%CV. For calculation of inter-assay %CV, the antibody concentrations observed in the representative sera samples from three independent plate measurements were taken into consideration. The inter- assay %CV was found to be <9.6%.

### Assay validation with clinical sera samples

**Figure 6:**
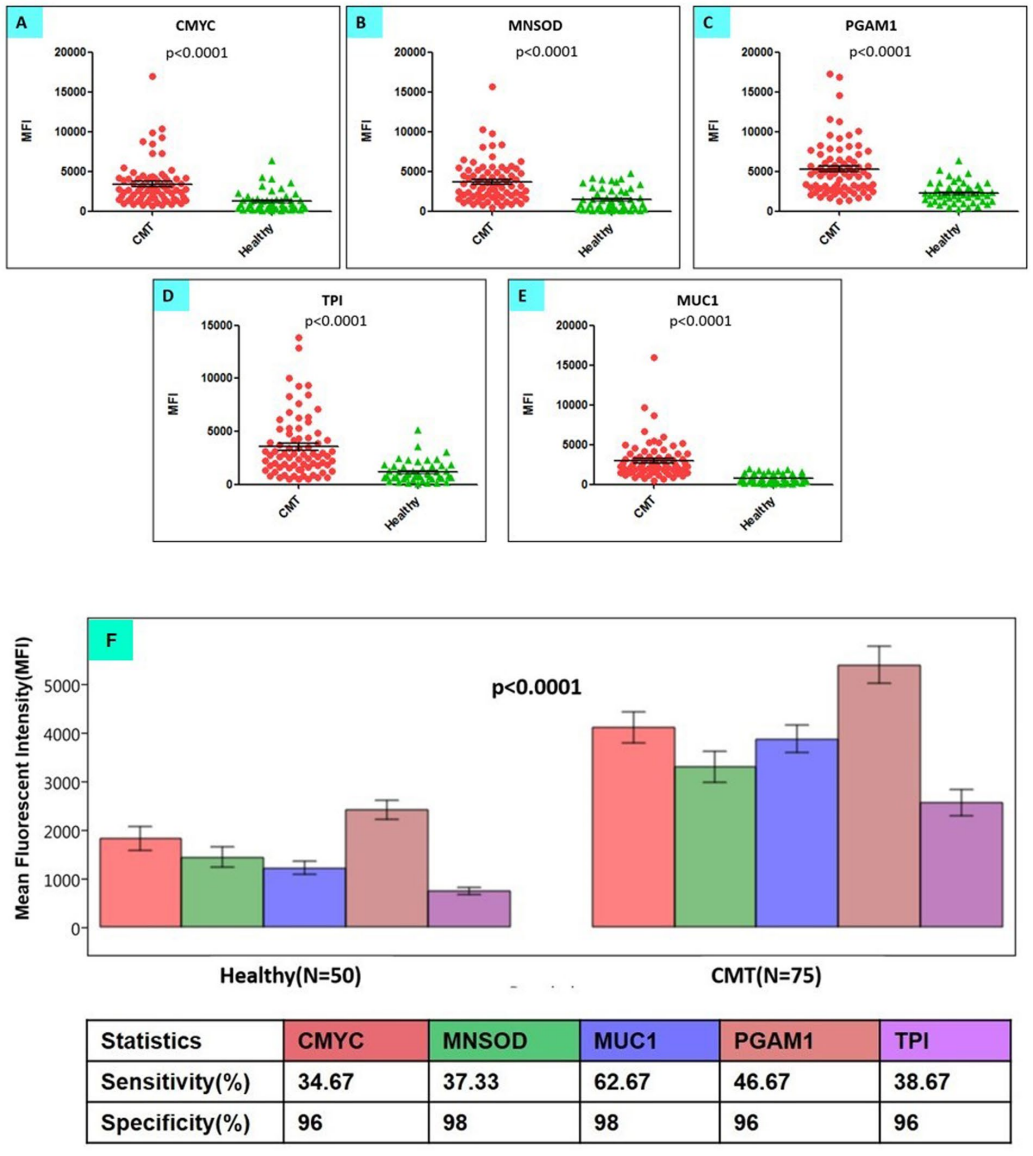
Comparison of MFI values corresponding to autoantibody response (against five TAAs) in 50 healthy and 75 canine mammary tumour sera. (**A**–**E**) Scatter diagrams representing CMYC, MNSOD, PGAM1, TPI & MUC1 autoantibody response in canine mammary tumour (CMT) sera (n = 75) and healthy dog sera (n = 50). (**F**) At 1:200 dilution of sera samples, the multiplex assay comprising of 5 autoantigens, namely TPI, PGAM1, MUC1, MNSOD, & CMYC coupled microspheres, produced significantly higher average MFI signals in CMT sera (n = 75) compared to healthy dog sera samples (n = 50) (Mann-Whitney, p value < 0.0001). At cut-off value (avg. MFI of healthy sera + 2 SD), individual assays were found to be highly specific (96–98% specificity). The bars represent average MFI values and error bars represent standard error.

**Figure 7:**
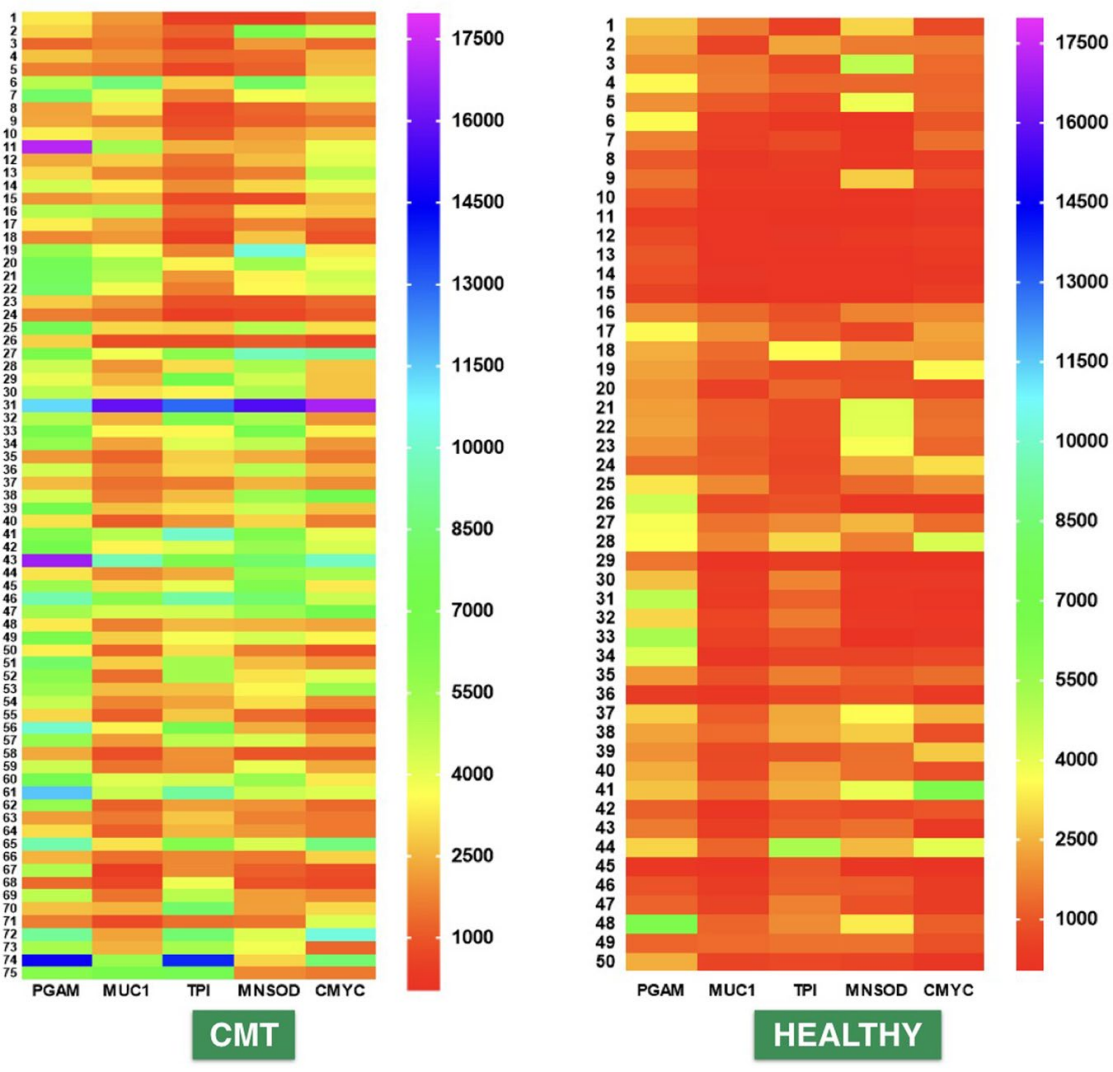
Heat maps of the MFI signals generated from CMT group and healthy group Comparison of heat maps of the MFI signals obtained from CMT (n = 75) and healthy dog sera (n = 50) by the multiplex assay revealed that majority of CMT sera have MFI values higher than 2,500 for all the five biomarkers, while most of the healthy dog sera have MFI values below 2,500. Heat map scale indicates the range of MFI values.

**Figure 8:**
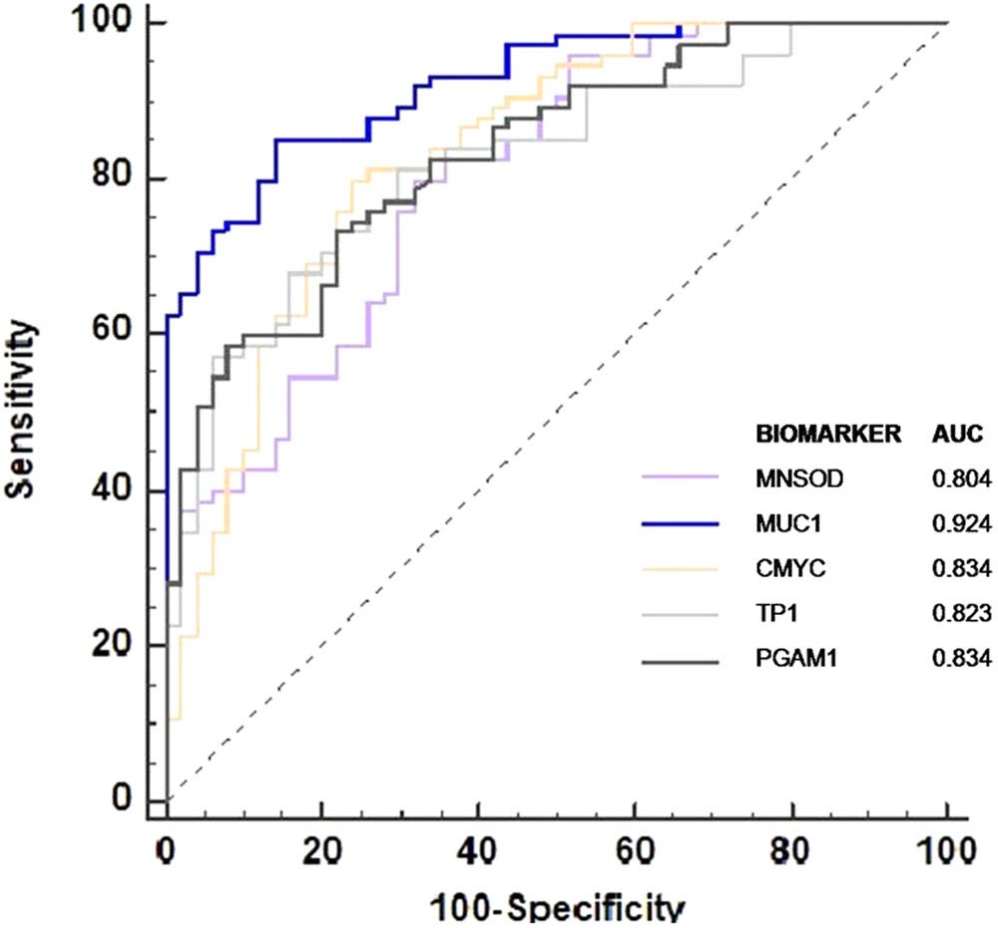
ROC analysis for individual autoantibody biomarkers: The area under the curve (AUC) for each individual autoantibody biomarker is more than 0.8 indicating the ability of individual assays to discriminate between CMT (n = 75) and healthy dog sera (n = 50). Comparison of ROC curves reveals maximum AUC for MUC1 biomarker followed by CMYC & PGAM1.

The validity of an assay is defined as its ability to distinguish between diseased and healthy individuals. Thus, to determine assay validity, the multiplex assay, comprising a panel of 5 candidate autoantigens, was used for screening of circulating autoantibodies in total 125 dog sera samples, including 75 canine mammary tumour (CMT) sera and 50 healthy dog sera. At 1:200 dilution of sera samples; TPI, PGAM1, MUC1, MNSOD, & CMYC coupled microspheres, produced significantly higher average MFI signals in CMT sera, as compared to healthy dog sera samples (Mann-Whitney, p value < 0.0001) (Fig. 6A–E). Taking the cut-off value as average MFI of healthy sera + 2 SD, the individual assays were found to be highly specific, with specificities ranging from 94% to 98%. However, the frequencies of autoantibodies to single TAA were relatively low, leading to lower sensitivities ranging from 34.6 to 60% for the individual TAAs (Fig. 6F). Among all TAA, sensitivity and specificity exhibited by MUC1 assay was found to be highest (Supplementary Tables S3–S7). Comparison of heat maps of the MFI signals generated from CMT and healthy dog sera revealed that majority of CMT sera have MFI values higher than 2,500 for all the five biomarkers, while most of the healthy dog sera have MFI values below 2,500 (Fig. 7). ROC curve analysis, showed that the area under the curve (AUC) of each TAA is more than 0.8 (Fig. 8), indicating the ability of individual assays to discriminate between tumour sera and healthy dog sera. Upon comparison of ROC curves, the maximum AUC (0.92) was observed with MUC1. There were significant differences in AUC of MUC1 and other autoantibody biomarkers (p < 0.05) indicating the usefulness of anti-MUC1 antibodies in detection of canine mammary tumour. The Pearson correlation coefficient, r was found to be greater than 0.5 for all biomarkers, indicating significant (p < 0.0001) correlation among different TAAs (Table 2). A maximum positive correlation coefficient (r) was observed between MNSOD and MUC1 biomarker (r = 0.75). Upon analysis of the expression patterns of these autoantibody biomarkers in different types (benign and malignant) and grades (I–III) of canine mammary tumours, it was observed that these 5 autoantibody biomarkers were present across all tumour grades and types. A significant positive correlation (r = 0.25, p < 0.05) was observed for presence of malignancy and TPI biomarker in clinical cases of canine mammary tumour. Among malignant CMTs, autoantibodies to TPI, MUC1, PGAM1, CMYC and MNSOD, were present across all tumour grades. MUC1 antibodies were found to be associated with 80% of grade III cancers as compared to 50% of early grade cancers (including grade I & II cancers), indicating their role in aggressiveness of canine mammary tumours.

**Table 2:** Correlation among different autoantibody biomarkers.

	MNSOD	PGAM1	MUC1	TPI	CMYC
MNSOD		0.547* 0.411–0.659**	0.752* 0.665–0.819**	0.576* 0.446–0.683**	0.728* 0.634–0.801**
PGAM1	0.547* 0.411–0.659**		0.713* 0.614–0.790**	0.677* 0.570–0.762**	0.620* 0.499–0.717**
MUC1	0.752* 0.665–0.819**	0.713* 0.614–0.790**		0.648* 0.533–0.739**	0.740* 0.649–0.810**
TPI	0.576* 0.446–0.683**	0.677* 0.570–0.762**	0.648* 0.533–0.739**		0.640* 0.524–0.733**
CMYC	0.728* 0.634–0.801**	0.620* 0.499–0.717**	0.7406* 0.649–0.810**	0.640* 0.524–0.733**	

[*Pearson correlation coeffient(r), ** 95% confidence interval for r].

### Evaluation of diagnostic utility of the autoantibody biomarker panel in canine mammary tumour immunodiagnosis

**Figure 9:**
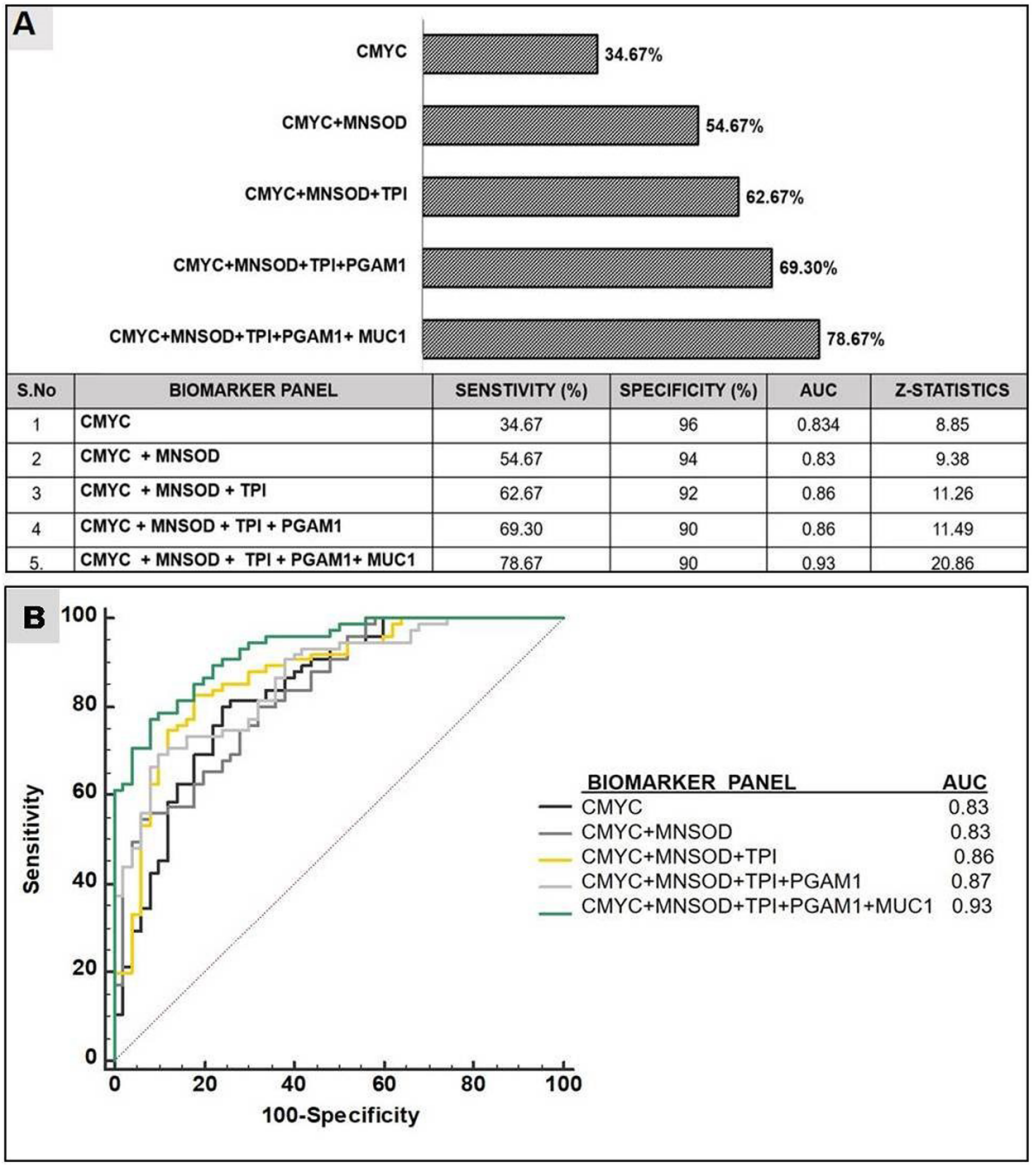
Diagnostic value of different autoantibody biomarker panels for canine mammary tumour: (**A**) With successive addition of TAAs to a total of 5 antigens in the multiplex assay, there is a stepwise increase in sensitivity. Out of 75 CMT sera samples analysed, 78.67% showed detectable level of autoantibodies cumulatively to any of these five TAAs, which was significantly higher than the frequency in sera from healthy individuals(n = 50) (p < 0.001). (**B**) Comparison of ROC curves reveals that with increase in the number of biomarkers in the panel, there is also an increase in AUC, with maximum AUC of 0.931(p < 0.0001) for the combined panel of five biomarkers.

Further, we determined the ability of the biomarker panel for detection of canine mammary tumours (CMTs). Out of 75 CMT sera samples analysed, 78.6% (59/75) had a detectable level of autoantibodies cumulatively to any of these five TAAs, which is significantly higher than the frequency in sera from healthy individuals (p < 0.001). The results depicted in Fig. 9A clearly establish that, with the successive addition of TAAs in the multiplex panel to a total of 5, there is a stepwise increase in sensitivity reaching upto 78.6%. The MFI scores generated by all TAAs were analysed by ROC curve, which provides an index for test’s accuracy by plotting the sensitivity against 1–specificity for each result value of the test. Upon analysis it was observed that AUC of the combined panel of five biomarkers was 0.931 (p < 0.0001), greater than AUC for individual biomarkers, demonstrating the strong discriminative power of panel of 5 biomarkers. Comparison of ROC curves reveals that with increase in the number of biomarkers in the panel, there is also an increase in AUC, with maximum AUC of 0.931(p < 0.0001) for the combined panel of five biomarkers (Fig. 9B). Considering the healthy average MFI + 2 SD as cut-off limit for individual biomarkers, the multiplex assay was found to be 78.6% sensitive and 90% specific (Table 3). Further, no healthy sample was positive for more than 1 autoantibody biomarker above the cut-off limits, whereas 60% (45/75) of the tumour sera samples were positive for more than 1 biomarker. Thus, assuming the presence of more than 1 biomarkers above the cut-off limit as a criterion for positivity, the five-plex assay was 100% specific and 60% sensitive. To further address the question of how valuable is the approach of antibody detection to a panel of five TAAs in separating dogs with and without mammary tumours, a group of parameters, such as Youden’s index, positive and negative predictive value (+PV/−PV), etc, were calculated and summarized in Table 3. The combined biomarker panel showed a positive predictive value of 87.88% and the negative predictive value of 71.19%. Taken together, these data show the usefulness of the multiplex assay in increasing the clinical diagnostic quality and value for mammary cancer diagnosis in dogs.

**Table 3:** Diagnostic efficacy parameters for panel of combined autoantibody biomarkers (PGAM1, CMYC, MUC1, TPI, and MNSOD).

ROC analysis for combined biomarker panel		
Statistics	Value	95% CI
Sensitivity	78.6%	66.21% to 86.21%
Specificity	90%	80.77% to 97.78%
AUC	0.931	0.870 to 0.968
z statistic	20.86	—
Youden index J	0.70	—
Positive Likelihood Ratio	4.83	2.53 to 9.23
Negative Likelihood Ratio	0.27	0.17 to 0.42
Positive Predictive Value	87.88%	79.15% to 93.26%
Negative Predictive Value	71.19%	61.52% to 79.24

## Discussion

Exploitation of the immunological responses evoked against tumour associated autoantigens (TAAs) is an emerging strategy for developing new tools for non-invasive detection of cancers. Assay based on demonstration of anti-TAA antibodies in sera of patients could be of great importance for early detection of cancer because detectable amount of antibodies against TAA are formed well before the tumour phenotype arises^2,52–58^. Further, detection of autoantibodies in sera of animals is more reliable than detection of TAAs, which are not always present in sera in detectable levels and are relatively unstable as they can degrade with time in comparison to antibodies^1,2,4^.

Mammary cancer results from the derailment of multiple cell signalling pathways and regulatory processes. Thus, by elucidation of a single biomarker, accurate diagnosis of the disease cannot be made and multiple biomarkers need to be identified. Research in the past few years have established the fact that multiplexing autoantibody biomarkers could lead to significant increase in sensitivity and specificity for cancer detection^24,25,50,58–61^. Therefore, the aim of this study was to develop a multiplex assay for detecting autoantibodies against a panel of five TAAs in clinical cases of canine mammary tumour, which is well established as a model for human breast cancer studies. Three TAAs, including MNSOD, TPI, and PGAM1, were selected as potential autoantigens for canine mammary tumour panel based on findings of Zamani-Ahmadmahmudi *et al*.^39^. Autoantibodies against other 2 autoantigens namely CMYC and MUC1 were selected as biomarkers for panel assay based on the performance of in-house developed ELISA in distinguishing canine mammary tumours from healthy controls (Supplementary Table S8).

The combined AUC for the panel of biomarkers used for magnetic bead based assay is 0.931 (p < 0.0001), which clearly reflects the ability of the five-plex assay in discriminating dog mammary tumour patients and healthy controls. Further, the assay could be conducted in 3 hours using only one microliter of serum sample and could detect clinical cases of dog mammary tumour with sensitivity and specificity of 78.6% and 90%, respectively. In this study, we have reported for the first time a multiplexed assay for detection of autoantibodies in canine tumours, utilizing luminex technology and halo-tag coupling strategy. An interesting feature of this study was that with an increase in the number of autoantibody biomarkers in the multiplex immunoassay, the likelihood of detecting antibody in the serum samples increased. The likelihood of detecting CMT (95% confidence interval for sensitivity) was 24.04–46.54% when only 1 biomarker (CMYC) was used, which increased to 66.21–86.21% when five biomarkers were used. Further, no healthy sample had more than 1 autoantibody biomarker above the cut-off limits, whereas 60% (45/75) of the tumour sera samples were positive for more than 1 biomarker. Thus, assuming the presence of more than one biomarker above the cut-off limit as a criterion for positivity, the five-plex assay is 100% specific and 60% sensitive. Similar results were observed in a human breast cancer study, where researchers have found that successive addition of TAAs to a total of six antigens, led to a stepwise increase in positive antibody reactions reaching a sensitivity of 67.3% and specificity of 92.2%^59–61^. Therefore, both the sensitivity and specificity of the assay could be improved by expanding the autoantibody panel to include more autoantibodies which might be more selectively associated with canine mammary tumour. For this, more autoantibody biomarker candidates associated with canine mammary tumour needs to be identified, as only a few autoantibody biomarkers have been reported so far in dogs. Several studies have reported that dog and human breast cancer share common tumour antigens. Therefore, based on leads from human cancer studies, we have identified autoantibodies to CMYC and MUC1 in canine tumours. In future, more efforts need to be diverted towards identification of autoantibody biomarkers in canines.

To conclude, the multiplex autoantibody assay holds great promise for canine mammary tumour diagnosis. To adapt the technique for mass screening, a detailed follow-up study needs to be conducted with more number of samples, with different tumour types and stages to further validate the performance of the autoantibodies across tumour histology and type. This multiplex luminex assay could be envisaged for screening of the high-risk population with subsequent confirmatory tests. Due to the similarity of tumour proteome profile in dogs with that of humans, the canine mammary tumour serves as an excellent model for studying human cancer biology and therapy. Therefore, further investigations with a number of samples are required to determine the efficacy of these serum biomarkers for early diagnosis or prognosis of canine, as well as, human mammary cancer.

## Electronic supplementary material

Supplementary Information

## Data Availability

All the relevant data pertaining to the study shall be made available upon request.
