# Supplementary material for: Multiplexed Autoantibody Signature for Serological Detection of Canine Mammary Tumours
Source: Sci Rep. 2018 Oct 25;8:15785. doi: 10.1038/s41598-018-34097-0 (PMC6202347; doi:10.1038/s41598-018-34097-0)
Supplement: Supplementary file 1 — Supplementary Information [file 41598_2018_34097_MOESM1_ESM.doc]

**Supplementary Information**

**Multiplexed Autoantibody Signature for Serological Detection of Canine Mammary Tumours**

Shahid Hussain^1#^, Sonal Saxena^1#^*, Sameer Shrivastava^1#^***,** Richa Arora^1^, Rajkumar James Singh^1^, Subas Chandra Jena^1^, Naveen Kumar^2^, Anil Kumar Sharma^3^, Monalisa Sahoo^3^, Ashok Kumar Tiwari^1^, Bishnu Prasad Mishra^1^, and Raj Kumar Singh^1^*

*Corresponding authors Email ID: [sonalvet@gmail.com](mailto:sonalvet@gmail.com), [sameer_vet@rediffmail.com](mailto:sameer_vet@rediffmail.com), [rks_virology@rediffmail.com](mailto:rks_virology@rediffmail.com)

^#^ Authors contributed equally (1^st^, 2^nd^ and 3^rd^ author)

^1^Division of Veterinary Biotechnology, ICAR-Indian Veterinary Research Institute [Deemed University] Izatnagar, Bareilly, UP, India

^2^Division of Veterinary Surgery, ICAR-Indian Veterinary Research Institute [Deemed University], Izatnagar, Bareilly, UP, India

^3^Division of Veterinary Pathology, ICAR-Indian Veterinary Research Institute [Deemed University], Izatnagar, Bareilly, UP, India

**Supplementary figures**


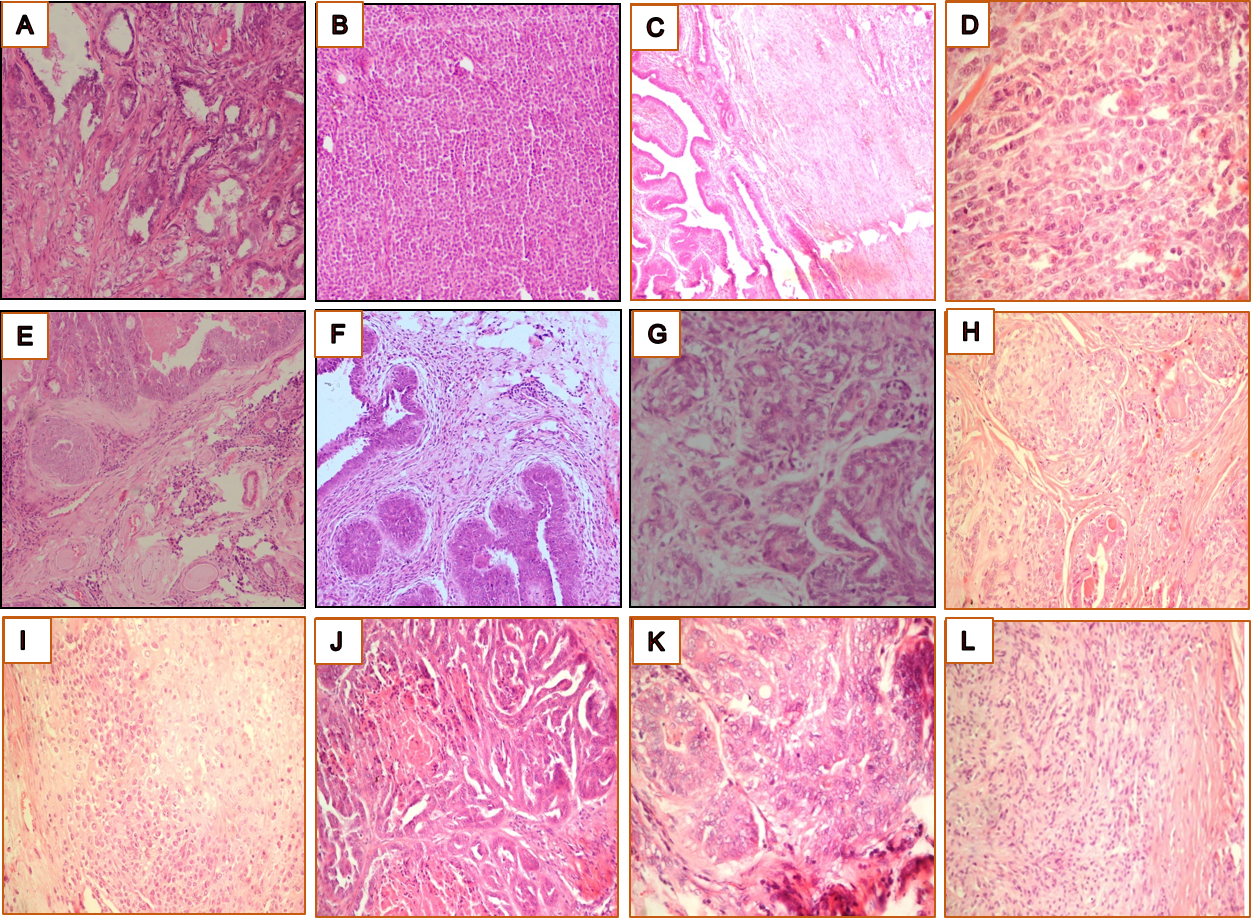


**Supplementary Figure S1**: **Hematoxylin & eosin stained malignant and benign mammary tumour tissue sections.** (A) Mixed myoepithelioma (B) Invasive solid carcinoma, (C) Carcinosarcoma, (D) Solid carcinoma, (E) Mixed mammary capillary cystic adenocarcinoma, (F) Complex carcinoma (G) Complex carcinoma, (H) Adenomyoepithelioma, (I) Malignant mixed mammary tumour, (J) Tubullo papillary carcinoma, (K) Adenoma, (L) Fibroma


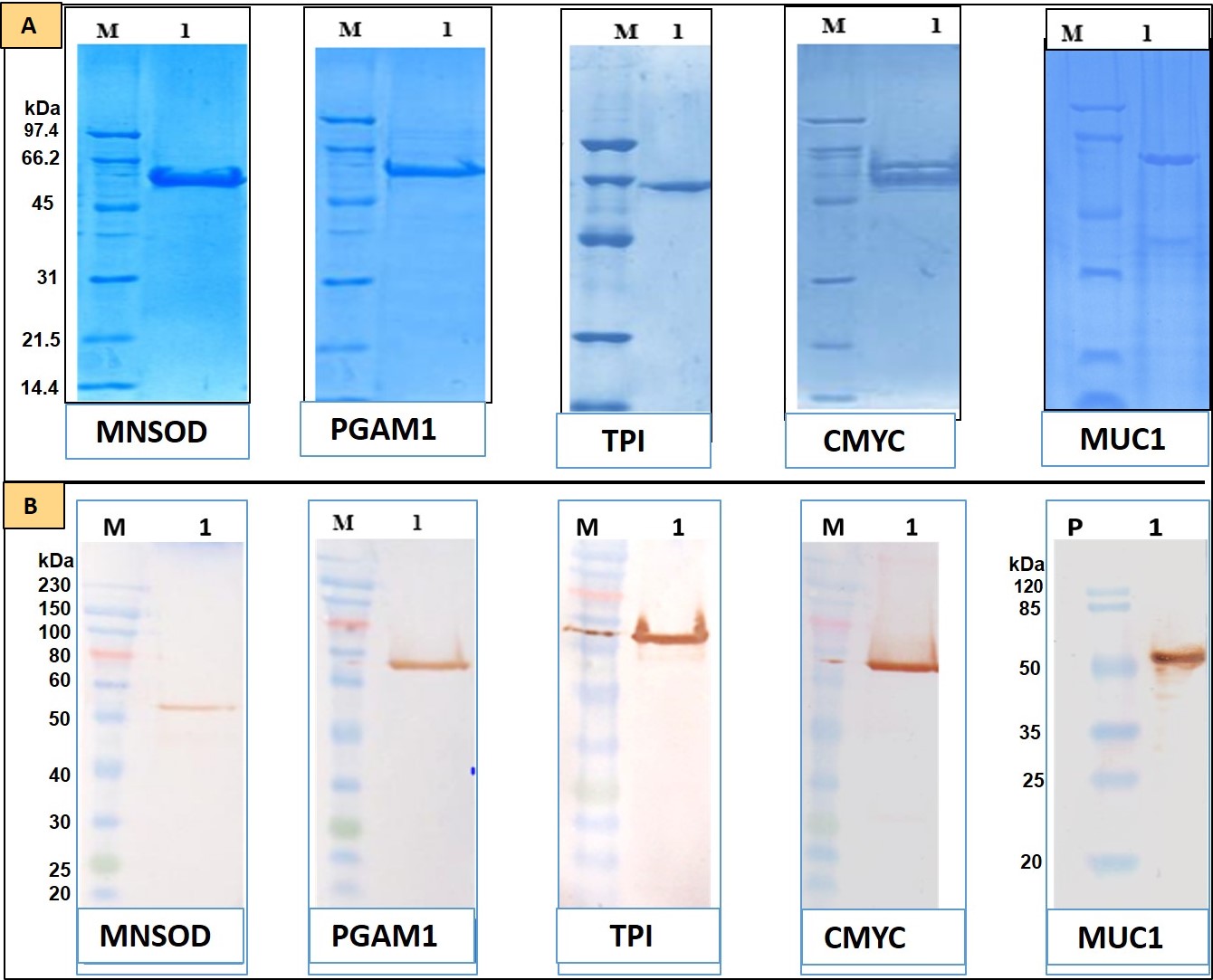


**Supplementary Figure S2: SDS-PAGE and Western blot characterization of halotag fused recombinant MNSOD, PGAM1, TPI, CMYC & MUC1. (A) SDS PAGE analysis of recombinant MNSOD, PGAM1, TPI, CMYC and MUC1**. Lane M, Unstained protein molecular weight marker; Lane1, Purified recombinant protein. (B) Western blot characterization of recombinant MNSOD, PGAM1, TPI, CMYC and MUC1 using commercial polyclonal antibodies against corresponding proteins. Lane M, Colourplus prestained protein molecular weight marker (NEB); Lane P, Pierce prestained protein molecular weight marker (Thermofischer); Lane1, Immunoreactivity of the purified recombinant protein with the corresponding antibodies.


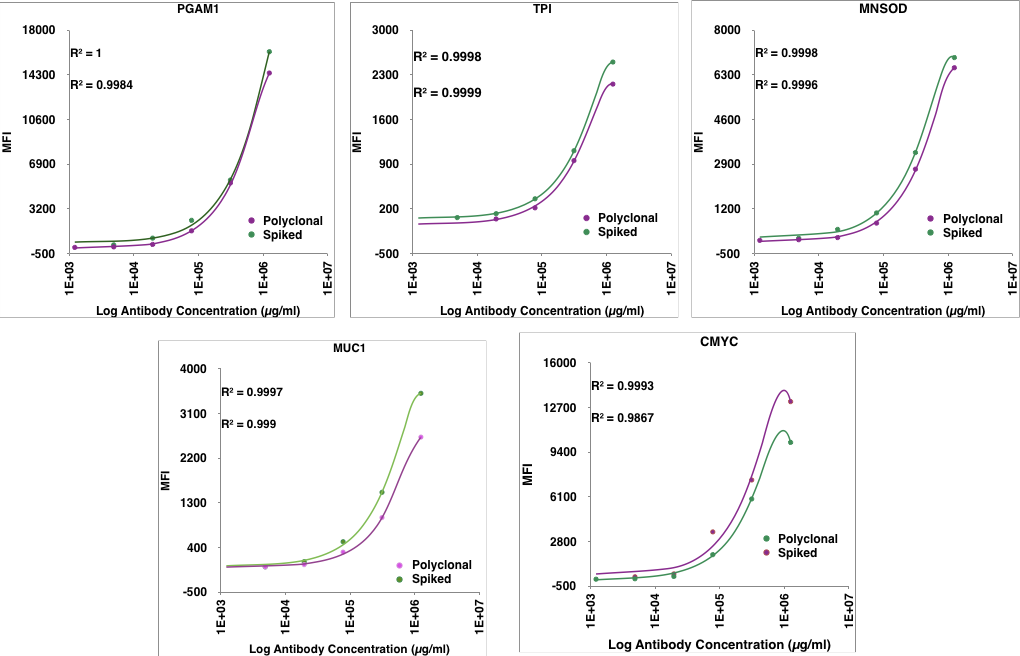


**Supplementary Figure S3 : Comparison of concentration response curve of the spiked sera and standard polyclonal sera to ascertain assay parallelism.** Standard polyclonal antibodies were spiked in a healthy dog sera. Ten-fold serial dilutions of standard polyclonal sera and spiked sera were prepared and concentration response curves were compared using four-parameter logistic (4-PL) curve fitting to ascertain assay parallelism

**Supplementary Table S**1: Histopathological details of canine mammary tumour (CMT) cases used for the study

| Case No | Histopathology | Malignancy | Grade | Case No | Histopathology | Malignancy | Grade |
| --- | --- | --- | --- | --- | --- | --- | --- |
| 1 | Adenoma | Benign | - | 31 | Carcinosarcoma | Malignant | II |
| 2 | Fibroadenoma | Benign | - | 32 | Solid carcinoma | Malignant | II |
| 3 | adenomyoepithelioma | Benign | - | 33 | Early carcinoma | Malignant | II |
| 4 | Mixed myoepithelioma | Benign | - | 34 | Early invasive ductal carcinoma | Malignant | II |
| 5 | Fibrocystic adenoma | Benign | - | 35 | Anaplastic carcinoma | Malignant | II |
| 6 | Fibroma | Benign | - | 36 | Carcinomasarcoma | Malignant | II |
| 7 | Fibroadenoma | Benign | - | 37 | Tubulloacinar carcinoma | Malignant | II |
| 8 | Cystic papillary adenoma | Benign | - | 38 | Complex carcinoma | Malignant | II |
| 9 | Papillary Adenoma | Benign | - | 39 | Tubullopapillary carcinoma | Malignant | II |
| 10 | Fibroadenoma | Benign | - | 41 | Carcinosarcoma | Malignant | II |
| 11 | Adenosquamous carcinoma | Malignant | I | 42 | Complex carcinoma | Malignant | II |
| 12 | Fibrosarcoma | Malignant | I | 43 | Carcinosarcoma | Malignant | II |
| 13 | Tubular adenocarcinoma | Malignant | I | 44 | Tubulloacinar simple carcinoma | Malignant | II |
| 14 | Early simple carcinoma | Malignant | I | 45 | Complex carcinoma | Malignant | II |
| 15 | Adenosquamous cell carcinoma | Malignant | I | 46 | Complex carcinoma | Malignant | II |
| 16 | Tubular carcinoma | Malignant | I | 47 | Cystic papillary ductular carcinoma | Malignant | II |
| 17 | Malignant Hemangioma | Malignant | I | 48 | Mixed mammary tumour | Malignant | II |
| 18 | Solid carcinoma | Malignant | I | 49 | Tubuloacinar solid carcinoma | Malignant | II |
| 19 | Early cystic adenocarcinoma | Malignant | I | 50 | Solid adenocarcinoma | Malignant | II |
| 20 | Mast cell tumour | Malignant | I | 51 | Invasive solid carcinoma | Malignant | III |
| 21 | Complex carcinoma | Malignant | I | 52 | Complex carcinoma | Malignant | III |
| 22 | Mast cell tumour | Malignant | I | 53 | Solid carcinoma | Malignant | III |
| 23 | Complex carcinoma | Malignant | I | 54 | Malignant mixed mammary tumour | Malignant | III |
| 24 | Early invasive carcinoma | Malignant | I | 55 | Basal cell carcinoma | Malignant | III |
| 25 | Tubullopapillary Adenocarcinoma | Malignant | II | 56 | Simple solid carcinoma | Malignant | III |
| 26 | Lipid rich carcinoma | Malignant | II | 57 | Osteochondrosarcoma | Malignant | III |
| 27 | Tubular carcinoma | Malignant | II | 58 | Squamous cell carcinoma | Malignant | III |
| 28 | Lipid rich carcinoma | Malignant | II | 59 | Solid carcinoma | Malignant | III |
| 29 | Early carcinoma | Malignant | II | 60 | Squamous cell carcinoma | Malignant | III |
| 30 | Tubulloacinar simple carcinoma | Malignant | II | 61-75 | Mammary tumours confirmed by FNAC* | - | - |

**Supplementary Table S2: Primer sequences used for amplification of target genes**

| **Gene name** | **Primer** | **Sequence (5’-3’)** |
| --- | --- | --- |
| **TPI** | Forward | G ACCTTC AGTGTGTCGCTC |
|  | Reverse | GG AAT AGG GGA GATGGA TG |
| **PGAM1** | Forward | GCCA TGGCCG CGT ACA AG |
|  | Reverse | ATCCTCAGGA AAG CAA TC |
| **CMYC** | Forward | GAGA CACCGC CCA CCAC |
|  | Reverse | TTAGGCACCA GAG TTCC |
| **MNSOD** | Forward | GCGCTCA CCATGT TGTC |
|  | Reverse | TGAAAACCTTATTTTGCTC |
| **MUC1** | Forward | AATCCCAGCAGCAACTACTAC |
|  | Reverse | GGTTCGGGTTCATGTAAGAGAG |

**Supplementary Table S**3: Diagnostic efficacy parameters for PGAM1 autoantibody biomarker assay

|  | | |
| --- | --- | --- |
| Statistics | Value | 95% CI |
| Sensitivity | 46.67% | 35.05% to 58.55% |
| Specificity | 96.00 % | 86.29% to 99.51% |
| Cutoff value(Healthy Avg± 2 SD) | 4894.626 | - |
| AUC | 0.834± 0.0351 | 0.757 to 0.895 |
| z statistic | 9.503 | - |
| Youden index J | 0.5133 | - |
| Positive Likelihood Ratio | 11.67 | 2.94 to 46.34 |
| Negative Likelihood Ratio | 0.56 | 0.45 to 0.69 |
| Positive Predictive Value | 94.59% | 81.50% to 98.58% |
| Negative Predictive Value | 54.55 % | 49.08% to 59.90% |

**Supplementary Table S**4: Diagnostic efficacy parameters for MNSOD autoantibody biomarker assay

| Statistics | Value | 95% CI |
| --- | --- | --- |
| Sensitivity | 37.33% | 26.43% to 49.27% |
| Specificity | 98.00 % | 89.35% to 99.95% |
| Cutoff value (Healthy Avg± 2 SD) | 3782.15 | - |
| AUC | 0.804± 0.0393 | 0.723 to 0.870 |
| z statistic | 7.726 | - |
| Youden index J | 0.4800 | - |
| Positive Likelihood Ratio | 18.67 | 2.62 to 132.83 |
| Negative Likelihood Ratio | 0.64 | 0.53 to 0.76 |
| Positive Predictive Value | 96.55% | 79.74% to 99.50% |
| Negative Predictive Value | 51.04 % | 46.57% to 55.50% |

**Supplementary Table S5: Diagnostic efficacy parameters for MUC1 autoantibody biomarker assay**

| Statistics | Value | 95% CI |
| --- | --- | --- |
| Sensitivity | 62.67% | 50.73% to 73.57% |
| Specificity | 98.00 % | 89.35% to 99.95% |
| Cutoff value(Avg± 2 SD) | 1915.4 | - |
| AUC | 0.924±0.0219 | 0.862 to 0.964 |
| z statistic | 19.372 | - |
| Youden index J | 0.7133 | - |
| Positive Likelihood Ratio | 31.33 | 4.47 to 219.82 |
| Negative Likelihood Ratio | 0.38 | 0.28 to 0.51 |
| Positive Predictive Value | 97.92% | 87.01% to 99.70% |
| Negative Predictive Value | 63.64 % | 56.56% to 70.17% |

**Supplementary Table S6: Diagnostic efficacy parameters for TPI autoantibody biomarker assay**

| Statistics | Value | 95% CI |
| --- | --- | --- |
| Sensitivity | 38.67% | 27.64% to 50.62% |
| Specificity | 96.00 % | 86.29% to 99.51% |
| Cutoff value(Avg± 2 SD) | 4207.08 | - |
| AUC | 0.823 ±0.0369 | 0.745 to 0.886 |
| z statistic | 8.773 | - |
| Youden index J | 0.520 | - |
| Positive Likelihood Ratio | 9.67 | 2.41 to 38.71 |
| Negative Likelihood Ratio | 0.64 | 0.53 to 0.77 |
| Positive Predictive Value | 93.55% | 78.36% to 98.31% |
| Negative Predictive Value | 51.06 % | 46.36% to 55.75% |

**Supplementary Table S7: Diagnostic efficacy parameters for CMYC autoantibody biomarker**

| Statistics | Value | 95% CI |
| --- | --- | --- |
| Sensitivity | 34.67 % | 24.04 % to 46.54 % |
| Specificity | 96.00 % | 86.29 % to 99.51 % |
| Cutoff value(Avg± 2 SD) | 3135.38 | - |
| AUC | 0.834± 0.0378 | 0.757 to 0.895 |
| z statistic | 8.851 | - |
| Youden index J | 0.560 | - |
| Positive Likelihood Ratio | 8.67 | 2.15 to 34.90 |
| Negative Likelihood Ratio | 0.68 | 0.57 to 0.81 |
| Positive Predictive Value | 92.86% | 76.35% to 98.13% |
| Negative Predictive Value | 49.48 % | 45.14 % to 53.83% |

**Supplementary Table S8: Diagnostic potential of TPI, MNSOD, PGAM1, MUC1 & CMYC autoantibodies in canine mammary tumours**

Based upon human and canine breast cancer studies and literature available, 11 mammary tumour associated autoantigens were identified and autoantibodies to these 11 TAAs were screened by I-ELISA in clinical cases of canine mammary tumours(n=40) and healthy dogs(n=50). Out of 11 selected TAAs, autoantibodies to MUC1, CMYC, TPI, PGAM1, and MNSOD were present in higher frequency in dog mammary cancer sera than healthy sera.

|  | **Biomarker** | **Sensitivity** | **Specificity** |
| --- | --- | --- | --- |
| **1** | ERBB2/HER2/neu | 32.6 | 97 |
| **2** | TPI | 40.4 | 96 |
| **3** | PGAM1 | 50.5 | 96 |
| **4** | MUCIN 1 | 59.1 | 97 |
| **5** | YKL40/CHI3L1 | 24.6 | 91 |
| **6** | C-MYC | 38.2 | 97 |
| **7** | MnSOD | 42.0 | 97 |
| **8** | SURVIVIN/BIRC5 | 31.9 | 95 |
| **9** | MMP9 | 34.09 | 97 |
| **10** | MMP2 | 30.4 | 90 |
| **11** | HSP90 | 20.1 | 97 |
